# Adherence to the Planetary Health Diet and Its Association with Diet Quality and Environmental Outcomes in Croatian University Students: A Cross-Sectional Study

**DOI:** 10.3390/nu17111850

**Published:** 2025-05-29

**Authors:** Gordana Kenđel Jovanović, Greta Krešić, Elena Dujmić, Sandra Pavičić Žeželj

**Affiliations:** 1 Department of Health Ecology, Teaching Institute of Public Health of Primorsko-Goranska County, 51000 Rijeka, Croatia; sandrapz@uniri.hr; 2 Department of Health Ecology, Faculty of Medicine, University of Rijeka, 51000 Rijeka, Croatia; 3 Department of Food and Nutrition, Faculty of Tourism and Hospitality Management, University of Rijeka, 51410 Opatija, Croatia; gretak@fthm.hr (G.K.);

**Keywords:** diet quality, environmental impact, planetary health diet, students, sustainable nutrition

## Abstract

Background/Objectives: University students are at a critical life stage in terms of establishing lifelong dietary habits, yet little is known about the sustainability of their diets, especially in Croatia. This study aimed to assess the sustainability and environmental impacts of university students’ dietary patterns at the University of Rijeka using the Planetary Health Diet Index (PHDI) and to explore the associations with demographic, lifestyle, nutritional, and environmental variables. Methods: A cross-sectional study was conducted from October 2023 to March 2024 among 224 students (54% male, mean age 22.7 ± 2.2 years). Data collection included sociodemographic information, physical activity, and dietary intake (semi-quantitative FFQ). Diet quality was assessed using the PHDI, Mediterranean Diet Score (MDS), and Dietary Inflammatory Index. Environmental impact indicators (carbon, water, and ecological footprints) were calculated using energy-adjusted intake data and standardized life cycle assessment data. Results: Students exhibited moderate adherence to the Planetary Health Diet (mean PHDI: 55.5). Higher PHDI scores were significantly associated with vigorous physical activity, higher MDS, and anti-inflammatory dietary patterns (all *p* < 0.001). Despite male students showing slightly higher PHDI scores, their diets had significantly greater environmental impacts. A one-point increase in the PHDI correlated with smaller environmental footprints (carbon: β = −7.94; water: β = −13.88; ecological: β = −3.15; all *p* < 0.001), with a significant decrease observed particularly in the lowest- and highest-adherence groups, while no consistent or significant effects were found in the intermediate groups. The nutrient and food group analysis supported the health-promoting profile of diets aligned with the PHDI. Conclusions: This study highlights the moderate sustainability of students’ diets, with significant associations between diet quality and environmental impacts. University settings present key opportunities for the promotion of sustainable, health-oriented eating behaviors among young adults.

## 1. Introduction

Modern dietary patterns are becoming increasingly harmful to human and planetary health, significantly contributing to the global burden of disease and mortality. Non-communicable diseases (NCDs), including cardiovascular disease, obesity, and type 2 diabetes, are closely associated with diets low in fruits, vegetables, whole grains, and polyunsaturated fats and high in sodium, red and processed meats, and sugar-sweetened beverages [1,2,3,4]. In 2021 alone, NCDs accounted for 43.8 million deaths globally, with dietary risks contributing to 7.22 million of these, while, in Europe, a poor diet remains the second-leading cause of cardiovascular mortality [4,5,6]. At the same time, the current food system is a major driver of environmental degradation. Agri-food systems are major contributors to climate change and are exceeding the planetary boundaries [1,2,7]. They contribute to approximately one-third of anthropogenic greenhouse gas emissions, occupy over one-third of the global land area, and consume more than 70% of freshwater resources [7,8,9]. The environmental footprint of food production, combined with the high prevalence of diet-related diseases, results in substantial hidden costs that are not reflected in market prices, such as externalities that challenge the sustainability of global food systems [10]. Given the connection between human and environmental health, there is a growing emphasis on promoting sustainable diets, defined by the FAO [11] as those that have low environmental impacts while supporting nutritional adequacy and health for present and future generations. These diets must consider not only environmental sustainability but also cultural, social, and economic dimensions.

The EAT-Lancet Commission’s Planetary Health Diet (PHD) proposes a predominantly plant-based dietary pattern with limited animal-sourced and processed foods, aiming to align global eating habits with both health and environmental goals [2]. To quantify adherence to this model, the Planetary Health Diet Index (PHDI) was developed, offering a validated tool to evaluate the quality and sustainability of individual dietary patterns [12]. Aleksandrowicz et al. [13] provided evidence that adopting sustainable dietary patterns can lead to significant reductions in greenhouse gas emissions, land use, and water consumption and, at the same time, offer health benefits.

University students are at a life stage when long-term dietary habits are often established [14,15]. However, students frequently adopt unhealthy eating behaviors due to shared environments, limited food options, and lifestyle constraints [15,16]. The pattern of poor diet quality and low adherence to the Mediterranean diet among Croatian students is consistent with trends observed in other Mediterranean and non-Mediterranean European countries, where moderate or poor adherence is common and tends to decrease with age and during stressful periods, such as the COVID-19 pandemic [17,18,19]. Currently, there is limited knowledge regarding the sustainability of university students’ diets. Recent studies indicate that university students generally have moderate diet quality and environmental impacts, showing that higher diet quality is often associated with more sustainable eating behaviors [20,21,22,23,24,25,26,27]. Moreover, the sustainability of university students’ diets is challenged by poor diet quality, unhealthy food environments, and socioeconomic challenges. These indicate that the relationship between diet quality and environmental impacts is complex, as not all nutritionally optimal diets are environmentally sustainable, which highlights the need for integrated educational nutritional strategies to promote both health and sustainability among university students [16,23]. At the same time, university settings may offer opportunities to influence dietary patterns through education, food service policies, and campus initiatives [16,27]. Examining how closely student diets align with the PHD can provide insights into potential health and environmental benefits and inform strategies to promote sustainable eating among young adults. While there are initiatives concerning the sustainability and environmental impacts of diets in Croatia, currently, there are no peer-reviewed studies that specifically quantify the environmental impacts of dietary patterns.

Therefore, this study aimed to assess the sustainability and environmental impacts of the dietary patterns of university students of the University of Rijeka, Croatia, using the Planetary Health Diet Index, and to evaluate the associations between demographic, lifestyle, nutritional, and environmental impact variables. By assessing the relationship between diet quality, sustainability, and environmental impacts among young adults, this study increases the evidence base necessary to inform integrated strategies for health promotion and environmental management within higher education institutions in Croatia.

## 2. Materials and Methods

### 2.1. Procedure

This cross-sectional study was conducted between October 2023 and March 2024 and included university students from the University of Rijeka, Croatia. All participants were thoroughly informed about the study objectives and procedures and provided written informed consent before enrolment. This study was conducted following the Declaration of Helsinki, and the study protocol was approved by the Ethics Committee of the Teaching Institute of Public Health of Primorsko-Goranska County (Approval No.: 04-400-139/2-22). The survey instrument comprised three distinct sections. The first section collected data on participants’ sociodemographic characteristics through a series of structured questions. The second section used the standardized International Physical Activity Questionnaire—Long Form (IPAQ-LF) [28] to assess physical activity levels across multiple domains, including occupational, transportation, household, and leisure-time activities. The third section was a semi-quantitative Food Frequency Questionnaire (FFQ) designed to evaluate participants’ dietary intake over the preceding week [29]. The survey was conducted at the Department of School Medicine, Teaching Institute of Public Health of Primorsko-Goranska County, Croatia.

### 2.2. Participants

This study involved 224 students (121 women and 103 men), aged 19 to 27 years, based on the study criteria. To determine the required sample size, an a priori power analysis was conducted using the G*Power 3 software (Version 3.1.9.4). The analysis was based on an expected medium effect size (f = 0.3), degrees of freedom (*df* = 5), significance level (α) of 0.05, and statistical power (1 − β) of 0.95. The calculation determined a minimum sample size of 220 participants. Eligible participants were current university students aged 18 to 28 years, those who did not follow a specific dietary regimen or eating pattern, and those who had no history of chronic illnesses such as cardiovascular disease, diabetes, or medically diagnosed food allergies or intolerances requiring dietary restrictions. Participants were excluded if they did not meet the inclusion criteria, reported daily energy intakes below 600 kcal or above 3500 kcal (as determined from dietary records collected in the study), or were pregnant or lactating.

### 2.3. Measures

#### 2.3.1. Participants’ Characteristics

Sociodemographic characteristics collected in the survey included age, gender, university status, data on faculty affiliation, current field of study, and smoking status. The physical activity level was assessed using the self-administered International Physical Activity Questionnaire—Long Form (IPAQ-LF) [28]. The total physical activity score was calculated by multiplying the duration (in minutes) and frequency (days per week) of each activity type by its corresponding metabolic equivalent of task (MET) value and then summing the MET minutes/week for all activity domains, including vigorous, moderate, and walking activities. Participants were then categorized into one of three physical activity levels—vigorous, moderate, or low—based on the following IPAQ scoring criteria [28].

Vigorous: Engaging in vigorous-intensity activity on at least three days per week, accumulating a minimum of 1500 MET minutes/week, or participation in any combination of walking or moderate- or vigorous-intensity activities on seven or more days, achieving at least 3000 MET minutes/week.

Moderate: Engaging in vigorous-intensity activity for at least 20 min per day on three or more days, moderate-intensity activity or walking for at least 30 min per day on five or more days, or any combination of activities on five or more days, summing at least 600 MET minutes/week.

Low: Participants not meeting the criteria for either the moderate or vigorous categories were classified as having low physical activity.

#### 2.3.2. Anthropometric Measurements

Trained nursing personnel measured participants’ heights and body weights using a calibrated and validated scale with an integrated stadiometer (Vogel Halke & Seca, serial number 4569, Hamburg, Germany) under standardized conditions. The body mass index (BMI) was subsequently calculated. The body weight and body composition parameters were measured using a Tanita RD-545 Segmental Body Composition Analyzer (Tanita Corporation, n.d., Tokyo, Japan), but these data were not included in further analyses.

#### 2.3.3. Dietary Assessment

Food intake was assessed using a 98-item semi-quantitative Food Frequency Questionnaire (FFQ). Participants reported how often they consumed each food item over the past seven days. The FFQ provided frequency options ranging from “never or less than once per week” to “two or more times per day”, with a standard portion size for each listed food or drink item. Participants indicated whether their usual portion was smaller than, similar to, or larger than the standard portion. Daily intake (in grams per day) was then estimated by multiplying the reported consumption frequency by the adjusted portion size. Dietary energy and nutrient intakes were calculated using the Croatian Food Composition Database [30], while certain nutrients needed for the calculation of the Dietary Inflammatory Index were taken from the Danish [31] and American [32] databases.

#### 2.3.4. The Planetary Health Diet Index

Adherence to the EAT-Lancet Commission’s dietary recommendations was assessed using the Planetary Health Diet Index (PHDI), developed by Cacau et al. [12]. The PHDI consists of 16 components, each scored between 5 and 10 points, yielding a total score ranging from 0 to 150. Components with a maximum of 10 points include red meat, nuts and peanuts, legumes, poultry and substitutes, fish and seafood, eggs, fruits, vegetables, whole grains, dairy products, unsaturated fats, animal fats, and added sugars. Two additional components were the ratio of dark green leafy vegetables to other vegetables and the ratio of red and orange vegetables to other vegetables, which are each scored up to 5 points. Dietary intake for PHDI components was estimated based on the average consumption reported in the FFQ. Participants were then categorized into quartiles according to their total PHDI scores. The PHDI ranged from 20 to 100; therefore, the participants were divided into quartiles as follows: Quartile 1: PHDI 20–45; Quartile 2: PHDI 46–55; Quartile 3: PHDI 56–65; Quartile 4: PHDI 66–100.

#### 2.3.5. Mediterranean Diet Score

The Mediterranean diet was used as an indication of diet quality due to its well-established health benefits [33] and its recognition as a sustainable dietary pattern [34]. Adherence to the Mediterranean diet was assessed using the Mediterranean Diet Score (MDS), which is based on nine dietary components comprising both food groups and nutrient ratios. Healthy components, such as vegetables, fruits and nuts, cereals and tubers, legumes, fish, dairy products, and the ratio of unsaturated to saturated fats, were assigned 1 point if intake was above the gender-specific median and 0 points if below. In contrast, components considered less healthy, such as meat (including processed meat) and alcohol, were scored inversely, with 1 point given for consumption below the median. The total MDS ranges from 0 to 9 points, with adherence in this study classified as low (≤4 points) or high (≥5 points).

#### 2.3.6. Dietary Inflammatory Index

The Dietary Inflammatory Index (DII) was assessed in this study due to its ability to reflect the complex interactions among nutrients, bioactive compounds, and overall dietary patterns, rather than focusing solely on individual food components. Furthermore, the DII has been widely associated with a range of chronic disease outcomes, including all-cause mortality, depression, and intermediate risk factors such as elevated blood pressure and hypertension [35]. DII scores were calculated in this study using 42 of the possible 45 dietary components derived from participants’ average dietary intake, following the standardized protocol established by Shivappa et al. [36]. Briefly, for each dietary parameter, a z-score was computed by subtracting the global mean intake from the participants’ reported daily intake and dividing by the global standard deviation. These z-scores were then converted into centered percentiles and multiplied by the corresponding inflammatory effect score for each parameter. The resulting values were summed to generate an overall DII score for each participant. The DII score ranges from −8.87 (indicating a strongly anti-inflammatory diet) to +7.98 (indicating a strongly pro-inflammatory diet), with 0 representing neutral inflammatory potential. For this study analysis, participants were categorized into two groups: anti-inflammatory (DII ≤ 0) and pro-inflammatory (DII > 0).

#### 2.3.7. Environmental Impact Indicators

To evaluate the environmental impacts of participants’ diets, two publicly available life cycle assessment databases were applied. These databases provide comprehensive environmental impact data for a wide range of food items and are characterized by demanding data standardization protocols and multiple environmental indicators [37,38]. The environmental impact values from each database were integrated and matched to the quantity of each food, composite dish, and beverage item consumed by individual participants to estimate diet-related environmental burdens. The main environmental impact indicators assessed included the carbon footprint (expressed as kilograms of CO_2_ equivalents, kg CO_2_ eq), ecological footprint (m^2^·year), and water footprint (m^3^). Additionally, energy-adjusted environmental impact values were calculated to account for variations in total dietary intake, standardizing the environmental impact per 1000 kilocalories consumed.

### 2.4. Statistical Analysis

Statistical analyses were performed using TIBCO Statistica, v. 13.3.0 (TIBCO Software Inc., Palo Alto, CA, USA; 2017). The Kolmogorov–Smirnov test was used to verify the variables’ normality. Continuous variables were expressed as means and standard deviations, and categorical variables were expressed as absolute (n) and relative (%) frequencies. Participants were assigned to PHDI quartiles according to their scores (1st quartile: 20–45 points; 2nd quartile: 46–55 points; 3rd quartile: 56–65 points; 4th quartile: 66–100 points). The chi-squared test was used for comparisons of categorical variables, while Student’s *t*-test and ANOVA were used for continuous variables. All statistical tests were two-sided, and *p* values < 0.05 were considered statistically significant. The energy-adjusted method was used to adjust the usual nutrient intake for total energy intake, because nutrient consumption is associated with total energy intake, either because they contribute directly to energy intake or because individuals who consume more total energy also eat, on average, more of all specific nutrients [39]. This was also implemented for the environmental impact indicators and food components of the PHDI. To assess the associations between the PHDI and usual energy (kcal) and energy-adjusted nutrient and PHDI food component intakes, mixed-effects linear regression models were adjusted for potential confounders, i.e., gender (male vs. female), level of study (undergraduate vs. graduate), body mass index classification (underweight, normal weight, overweight, obesity), and physical activity level (low, moderate, vigorous). Similarly, an identical model to evaluate the association between the PHDI, the MDS, and the DII was used. For this analysis, the PHDI, the MDS, and the DII were standardized as z-scores, because crude scores have different scoring ranges (i.e., 0–150 vs. 0–9 points and vs.−8.87–7.98, respectively).

## 3. Results

Participants’ characteristics and those across PHDI quartiles, as well as the average PHDI scores, are presented in Table 1. A total of 224 students (54% men) participated in this study, with a mean age of 22.67 ± 2.19 years and a mean body mass index (BMI) of 24.11 ± 3.50 kg/m^2^. Men had slightly higher PHDI scores than women (56.96 vs. 53.87; *p* = 0.086). Graduate students (47%) had slightly higher PHDI scores compared to undergraduate students (56.27 vs. 55.83); however, these differences did not reach statistical significance (*p* = 0.188). A smoking status was reported by 25% of participants, and no significant difference was observed regarding the PHDI scores (smokers 54.64 vs. nonsmokers 54.87; *p* = 0.563). The BMI classification revealed that 28% of the participants were overweight and 7% were obese (*p* < 0.001). These groups showed slightly higher mean PHDI scores (overweight 56.56; obese 57.67), although the differences were not statistically significant (*p* = 0.750). Physical activity levels were significantly associated with the PHDI distribution (*p* < 0.001). Specifically, moderately active students (44%) had the lowest average PHDI scores (53.98; *p* = 0.101), while those engaging in vigorous physical activity had the highest scores (58.27), being statistically more likely to be placed in the higher PHDI quartiles (Q3 51%, Q4 53%; *p* = 0.001). The students had a mean MDS of 4.00, indicating moderate adherence to the MD, and a mean DII of 1.07, meaning that the average student diet had pro-inflammatory potential. An average PHDI score of 55.54 (range 20–100) indicated that the students had moderate adherence to the PHD. A significant proportion of students had poor adherence to the MD (63%; *p* < 0.001) and consumed pro-inflammatory diets, as indicated by DII scores greater than zero (62%; *p* < 0.001). High adherence to the MD (37% of all students) was significantly associated with higher PHDI scores (62.17 vs. 51.63 in low adherence; *p* < 0.001). Similarly, students with anti-inflammatory diets (DII < 0) showed significantly higher PHDI scores compared to those with pro-inflammatory diets (62.76 vs. 51.12, respectively; *p* < 0.001).

The mean PHDI across its quartiles was as follows: Q1 38.77 ± 7.52; Q2 52.85 ± 2.49; Q3 62.63 ± 2.52; and Q4 73.06 ± 6.31; *p* < 0.001. The quartile analysis based on the PHDI scores revealed significant differences across several variables. Only 17% of students were categorized into the highest quartile (Q4), with a mean PHDI score of 73.06, which was nearly double that of the lowest quartile (Q1, 38.77; *p* < 0.001). Male students were significantly represented in the second and third quartiles (Q2 57%, Q3 67%; *p* = 0.038), whereas undergraduate students predominantly were in the lowest quartiles (Q1 79%, Q2 51%; *p* = 0.004). Energy intake increased significantly across quartiles, reaching the highest value in Q3 (2692.25 ± 1135.83 kcal/day; *p* = 0.007). The MDS scores showed a consistent rising trend across PHDI quartiles (*p* < 0.001), paralleled by a larger proportion of students adhering to anti-inflammatory diets and showing greater adherence to the MD (*p* < 0.001). Conversely, poor adherence to the MD and pro-inflammatory dietary patterns were predominantly observed in the lower quartiles. The lowest quartile (Q1) had 86% of students with poor adherence to the MD and 93% of those with pro-inflammatory diets (Table 1).

**Figure 1:**
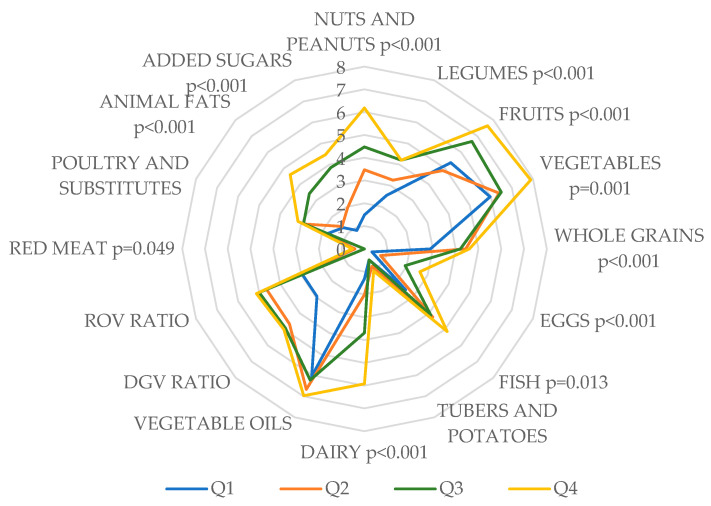
Average scores for the 16 Planetary Health Diet Index (PHDI) components among 224 Croatian university students across the PHDI quartiles. DGV, dark green vegetable ratio; ROV, red-to-orange vegetable ratio.

Figure 1 shows the average scores of the 16 PHDI components by quartile. The third and fourth quartiles had significantly higher scores for nuts and peanuts (*p* < 0.001), legumes (*p* < 0.001), fruits (*p* < 0.001), vegetables (*p* = 0.001), whole grains (*p* < 0.001), eggs (*p* < 0.001), fish (*p* = 0.013), dairy (*p* < 0.001), red meat (*p* = 0.049), animal fats (*p* < 0.001), and added sugars (*p* < 0.001). No significant differences were found in the scores for tubers and potatoes (*p* = 0.682), vegetable oils (*p* = 0.192), the dark green vegetable ratio (*p* = 0.191), the red-to-orange vegetable ratio (*p* = 0.191), or poultry and substitutes (*p* = 0.069).

Table 2 presents the means of the energy-adjusted environmental impact indicators regarding students’ demographic, lifestyle, and dietary factors and the quartiles of the Planetary Health Diet Index (PHDI). Men had significantly larger environmental footprints than women across all three indicators (carbon footprint *p* = 0.002; water footprint *p* = 0.017; ecological footprint *p* < 0.001). The level of study, BMI category, physical activity, and smoking showed weaker or no significant associations with the energy-adjusted environmental impact indicators. However, slight trends toward lower environmental impacts were observed among graduates and nonsmokers. Students with obesity showed the largest environmental footprints across all indicators, but the differences were not statistically significant (carbon footprint *p* = 0.176; water footprint *p* = 0.242; ecological footprint *p* = 0.278). Regarding physical activity, a slight U-shaped trend was observed, where moderately active students had a larger carbon footprint than students engaged in low or vigorous activities (*p* = 0.216). Students with a diet that more strongly adhered to the MD had a somewhat smaller water footprint, but this was only slightly significant (*p* = 0.052). There were no significant differences between pro-inflammatory and anti-inflammatory diets in relation to environmental impacts. Still, there was a clear and strongly inverse relationship between the PHDI score and all environmental impact indicators (all *p* < 0.001). The carbon footprint values decreased from 2.46 (Q1) to 2.01 (Q4), the water footprint decreased from 2.07 to 1.77, and the ecological footprint decreased from 5.43 to 4.35 (Table 2).

The energy-adjusted environmental impact indicators were analyzed in association with the Planetary Health Diet Index (PHDI) (Table 3). A one-point increase in the PHDI score was statistically associated with a smaller carbon footprint (β = −7.94, *p* < 0.001), water footprint (β = −13.88, *p* < 0.001), and ecological footprint (β = −3.15, *p* < 0.001), even after adjusting for gender, the level of study, the BMI category, and the physical activity level (Table 3). Specifically, each one-point increment in the PHDI was associated with a reduction of 7.94 kg CO_2_-eq in the carbon footprint, 13.88 m^3^ in the water footprint, and 3.15 m^3^ in the ecological footprint per 1000 kcal, after controlling for potential confounders. When examining the results by PHDI quartile, a one-point increase in the PHDI among students in the lowest-adherence group (Q1) was associated with a statistically significant decrease in the water footprint (β = −6.93, *p* = 0.012) and ecological footprint (β = −1.57, *p* = 0.038), as well as a marginally significant decrease in the carbon footprint (β = −3.76, *p* = 0.048). In contrast, a one-point increase in the PHDI of the highest-adherence group (Q4) showed significant decreases in both the carbon footprint (β = −4.72, *p* = 0.025) and water footprint (β = −7.98, *p* = 0.005), while the decrease in the ecological footprint did not reach statistical significance but showed a marginal trend (β = −2.79, *p* = 0.056). No statistically significant associations were observed for the second and third quartiles, and no consistent patterns of environmental impact were identified in these intermediate PHDI adherence groups (Table 3).

Additionally, an analysis was performed on the association between the PHDI and the average energy-adjusted nutrient intake in students’ diets. A one-point increase in the PHDI score was significantly associated with higher dietary intakes of energy (β = 2.15, *p* = 0.007), vegetable protein (β = 1.94, *p* < 0.001), monounsaturated fatty acids (β = 0.80, *p* < 0.001), polyunsaturated fatty acids (β = 12.47, *p* = 0.003), omega-3 fatty acids (β = 19.26, *p* < 0.001), dietary fiber (β = 2.59, *p* < 0.001), alcohol (β = 0.36, *p* = 0.020), folate (β = 0.13, *p* < 0.001), vitamin C (β = 0.19, *p* < 0.001), vitamin E (β = 1.57, *p* < 0.001), potassium (β = 0.02, *p* < 0.001), magnesium (β = 0.18, *p* < 0.001), and iron (β = 2.68, *p* < 0.001) (Table 4). Furthermore, flavonoid compounds showed statistically significant and positive associations with the PHDI, including flavan-3-ols (β = 0.15, *p* = 0.010), flavones (β = 7.16, *p* < 0.001), flavanols (β = 0.16, *p* < 0.001), flavanones (β = 0.11, *p* = 0.008), and anthocyanidins (β = 0.23, *p* = 0.004) (Table 4). Conversely, a unit increase in the PHDI score was significantly associated with lower intakes of total protein (β = −0.38, *p* = 0.017), animal protein (β = −0.46, *p* < 0.001), saturated fatty acids (β = −1.67, *p* < 0.001), omega-6 fatty acids (β = −39.76, *p* < 0.001), trans fats (β = −14.50, *p* < 0.001), and sodium (β = −0.01, *p* = 0.027). No significant associations were observed between the PHDI and other nutrients examined (Table 4).

Regarding food groups, higher PHDI scores were significantly associated with increased consumption of nuts and peanuts (β = 1.38, *p* < 0.001), legumes (β = 1.41, *p* = 0.005), fruits (β = 1.40, *p* < 0.001), vegetables (β = 3.56, *p* < 0.001), fish (β = 0.90, *p* < 0.001), and vegetable oils (β = 0.90, *p* < 0.001), as well as a higher MDS (β = 4.29, *p* < 0.001) (Table 5). On the other hand, a one-point increase in the PHDI was significantly associated with lower intakes of dairy products (β = −0.46, *p* < 0.001), poultry and meat substitutes (β = −0.90, *p* < 0.001), animal fats (β = −18.11, *p* < 0.001), and added sugars (β = −0.57, *p* < 0.001). A significant inverse association was also observed with the Dietary Inflammatory Index (β = −2.91, *p* < 0.001) (Table 5). This cross-sectional study found no significant associations between the overall PHDI scores and the intake of whole grains, eggs, tubers and potatoes, and red meat or the dark green vegetable ratio or red-to-orange vegetable ratio.

## 4. Discussion

This cross-sectional study examined the dietary patterns of 224 Croatian university students assessed with the Planetary Health Diet Index (PHDI) and focusing on its associations with demographic, lifestyle, nutritional, and environmental impact variables. The study results provide significant insights into the relationship between diet quality, environmental sustainability, and health-related behaviors among the young population. Both the Mediterranean Diet (MD) and the PHDI used in this study emphasize dietary patterns that promote health and sustainability. While the MD has verified specific health benefits and environmental advantages, the PHDI provides a structured basis for the assessment of adherence to sustainable dietary patterns across diverse populations. Integrating components from both approaches could give more insights and, in turn, enhance public health nutrition strategies specifically aimed at the young population.

The average diets of students had moderate alignment with the EAT-Lancet Planetary Health Diet (PHD), while every sixth student had a diet that strongly adhered to this environmentally friendly eating pattern. Male students’ diets had slightly stronger PHD characteristics than the diets of female students, although this difference was not statistically significant. This aligns with previous research indicating that gender differences in diet quality can be subtle and context-dependent [40]. Regardless of gender, university students tend to adopt similar unhealthy eating habits due to shared environments like campus cafeterias and limited cooking options [16,41]. Female university students, however, tend to have healthier eating habits and dietary patterns than male students, although unhealthy behaviors are common in both [16,42]. Subtle gender-specific differences in food choices and nutrient intake among students persist, indicating that the university setting only narrows gender differences, rather than creating distinctions [16,43]. Despite this, our study found that the dietary choices of male students were associated with a more unfavorable impact on the environment compared to the average dietary choices of female students. This study’s graduate students had diets that were more characteristic of the PHD than undergraduates, and these dietary patterns presented significantly greater environmental impacts. It seems that this difference may reflect greater nutritional knowledge or health consciousness among older students [44]. The PHDI has been found to vary according to lifestyle factors such as smoking status, with nonsmokers showing higher scores [12], which was observed in this study, albeit without a significant difference. Smoking status was not associated with the PHDI or environmental impact indicators, although studies of smoking and health behaviors often show that smokers have poorer health outcomes and lifestyle habits [45]. The findings of this study are consistent with some studies suggesting that smoking and diet quality may be independent factors in young adults [45]. The physical activity level was significantly associated with the PHDI score, with vigorous activity associated with higher diet quality. This supports evidence that physically active individuals tend to adopt healthier dietary patterns, possibly due to greater health motivation [46]. Furthermore, highly active students tend to consume more food to meet their performance needs, which may help them to better adhere to the recommended food group intakes in the PHD, leading to higher PHDI scores. However, since the index was adjusted for energy intake, these higher scores indicate improved diet quality and balance rather than just greater food quantities. Obesity is generally recognized for its detrimental impact on both public health and environmental sustainability. Moreover, the dietary patterns that contribute to obesity typically do not align with the principles of the PHDI [12,47]. Several studies have reported an association between greater adherence to PHDI-aligned diets and a lower body mass index [48,49,50]. Interestingly, however, other studies [26,51] have reported higher compliance with sustainable dietary models among individuals classified as overweight or obese. Regarding these findings, the present study did not observe a statistically significant difference in the PHDI scores across BMI categories among university students. A third of them were overweight or obese, with slightly higher scores than other BMI categories, but the lack of significant differences suggests complex relationships between diet quality and body weight that require further longitudinal investigation [52]. It may also indicate the possibility that most students rely on the meals offered in the campus restaurant, meaning that diet quality could be independent of BMI status in young adults [53].

On average, students’ diets showed poor adherence to the MD, and, if the observed dietary patterns continue, they could increase the risks of cardiovascular disease, metabolic disorders, some cancers, and neurodegenerative conditions [33]. The pro-inflammatory dietary profile of the average student diet may also elevate the risks of hypertension, insulin resistance, depression, and overall mortality [35]. The strong positive associations between the PHDI and Mediterranean Diet Score, along with inverse associations with the Dietary Inflammatory Index, highlight the shared emphasis on sustainable and anti-inflammatory dietary components. Students with high adherence to the Mediterranean diet and more anti-inflammatory dietary patterns had significantly higher PHDI scores. These findings confirm that diets composed of plant-based foods are linked to improved nutrient profiles and health outcomes, supporting both human and planetary health principles. This is consistent with recent studies showing that Mediterranean-style diets contribute to both reduced inflammation and environmental impacts [2,33]. The results are also consistent with the findings of the EHU12/24 study [23], which reported that better nutritional quality was associated with healthier dietary patterns and a reduced carbon footprint, and the study by Arrazat et al. [25], who indicated that the environmental impact of the Mediterranean diet may be higher due to the increased consumption of certain resource-intensive foods, such as fish and dairy. Currently, several studies among university students also discuss the environmental impacts of student diets, with findings that are consistent with the trends observed in this study, particularly regarding gender and Mediterranean diet adherence [20,21,22].

Since both low MD adherence and pro-inflammatory diets have been linked to an increased risk of depression and poorer mental well-being, actions aimed at the improvement of students’ dietary patterns are particularly relevant for such populations, who are in a critical period of cognitive and emotional development [54,55]. Adopting anti-inflammatory diets, such as the Mediterranean diet, can offer protective benefits regarding mental health in university students, who are undergoing a critical stage of emotional and cognitive development, as well as preparing them for future roles as highly educated adults who positively influence their environment.

The positive associations between the PHDI and the intake of plant-based proteins, unsaturated fats, fiber, vitamins, minerals, and flavonoids underscore the nutritional benefits of diets aligned with planetary health principles. The inverse relationships with saturated fats, animal protein, trans fats, and sodium further emphasize the health-promoting nature of higher PHDI scores. These findings support the PHDI’s validity as a marker of both nutritional and environmental quality. This balance of nutrient intake suggests cardiometabolic benefits; reduced inflammation, as supported by the inverse association with the Dietary Inflammatory Index; and a dietary pattern that may be protective against non-communicable diseases. The observed nutrient patterns among students are consistent with those observed in Mediterranean and plant-based diets, which have been beneficially associated with reduced chronic disease risks [56,57]. Regarding food groups, students’ higher PHDI scores were associated with the adequate consumption of nuts, legumes, fruits, vegetables, whole grains, eggs, fish, dairy, animal fats, and added sugars. These observations are similar to those of a study conducted among the university population [26] and are consistent with findings from other recent studies of university students’ diets [23,25]. These dietary patterns reflect the emphasis of the PHD on plant-based foods and limited animal products, supporting both health and environmental sustainability [2]. Furthermore, the findings of this and similar studies reinforce the potential of the PHD model, not only as the basis for a sustainable diet but also as a strategy for chronic disease prevention.

Interestingly, the higher environmental impact indicators among the observed subgroups of students may reflect higher overall energy intake or specific food choices that have higher resource demands [58], although this was significant only among male students. The reduced intake of animal fats, dairy, and processed foods, which are resource-intensive and contribute more to greenhouse gas emissions and land use, was also highly associated with the EAT-Lancet Planetary Health Diet in this study. However, when adjusted for energy intake, higher PHDI scores were significantly associated with lower environmental impacts, indicating that diet quality improvements can reduce environmental burdens when controlling for the energy density of the diet. This finding aligns with previous research demonstrating that adopting the Planetary Health Diet can reduce the per-calorie environmental footprints [59]. Based on this study’s results, even small improvements in one’s diet can yield significant benefits in decreasing its environmental impacts.

### 4.1. Strengths and Limitations

This cross-sectional study gives valuable insights into the dietary patterns of Croatian university students and their sustainability and environmental impacts. To the best of the authors’ knowledge, this is the first research to achieve these objectives among Croatian students. It is also the first study to use the PHDI and DII to assess the quality of the diets of Croatian students, and it is the first to assess the ecological impacts of their diets. Based on the study’s findings, we highlight the potential for university settings to act as intervention sites for the promotion of sustainable and health-conscious dietary behaviors. This study has several limitations that should be acknowledged. First, its cross-sectional design prevents any interpretation of causality. Second, dietary intake data were self-reported and may have been subject to recall bias, potentially affecting the accuracy of the findings. However, this was minimized by the assistance of an educated researcher during the completion of the questionnaires. Moreover, students’ dietary patterns may be influenced by their eating arrangements, e.g., eating at home with parents, self-preparing meals, or mostly relying on university cafeterias. Although this potential confounding factor was not assessed in the present study, it requires further investigation in future research. Although the sample included students with varying levels of study, it was limited to university students, which may restrict the generalizability of the results to the general Croatian population. Furthermore, the environmental impact estimates were derived from dietary intake data using standardized life cycle assessment metrics, which may not fully account for individual-level variability in food sourcing and production practices. This study was conducted entirely among students attending the University of Rijeka; thus, the findings may not be representative of dietary patterns among students at other Croatian universities. These limitations underscore the need for further observational research involving a broader student population across multiple university institutions in Croatia. Such studies could provide a more comprehensive understanding of students’ dietary behaviors and inform specific strategies to improve both nutritional health and environmental sustainability among young adults.

### 4.2. Implications and Future Directions

The present study’s findings indicate a moderate level of adherence of the average student’s diet to the Planetary Health Diet, with significantly higher adherence observed among graduate students and those with higher levels of physical activity. The study confirmed the strong associations with health-promoting dietary patterns, as assessed with indices such as the Mediterranean Diet Score and the Dietary Inflammatory Index. However, gender-based differences were found, as male students’ diets tended to have a greater environmental impact. Importantly, higher diet quality was linked to reduced environmental burdens when adjusted for energy intake, strengthening the evidence for the human and planetary health benefits of sustainable diets. However, diets characterized by greater adherence to planetary health principles may still exhibit considerable variation in their environmental impacts, depending upon the specific food selections and patterns of consumption [60]. Implementing strategies in university settings, such as educational initiatives, food cafeteria improvements, and behavior change interventions, has the potential to deliver benefits for individual and planetary environmental health, which are both important for the future quality of life of the young population [61]. Future research should explore longitudinal relationships to clarify causal pathways between diet quality, health outcomes, and environmental impacts among university students. Furthermore, qualitative studies could investigate the barriers and facilitators in the adoption of the Planetary Health Diet among university students to inform tailored nutrition education programs. The observed complexity of diet–weight associations suggests that future research should explore the longitudinal outcomes of sustainable dietary patterns and the role of campus food environments. Additionally, the independent relationship between smoking and diet quality indicates the need for integrated health behavior interventions. Future studies should also investigate the causal pathways between dietary habits, nutritional knowledge, lifestyle behaviors including physical activity, and environmental sustainability in university student populations from more universities in Croatia. The findings also underscore the importance of creating supportive university environments that facilitate access to nutritious and environmentally sustainable food options [62]. Examples may include an increase in the availability and visibility of plant-based, locally sourced, nutrient-dense, and sustainably produced food options in cafeterias and dining places [63]. Furthermore, introducing sustainability and health labeling on food and meals may help students to identify environmentally friendly choices associated with the PHD and Mediterranean diet [63,64]. Moreover, encouraging student populations to use application tools for diet assessments could foster awareness and self-monitoring of the ecological footprints of their dietary choices [65]. To increase their awareness and empower students to make informed decisions, workshops and educational programs on sustainable cooking, meal planning, and the environmental impacts of food choices could also be applied. Finally, this study’s findings could inform university and national policies on student nutrition, sustainability goals, and chronic disease prevention strategies aligned with planetary health frameworks. Updating the national dietary guidelines to reflect the latest evidence regarding healthy and sustainable eating is important in improving health and reducing environmental impacts, which could complement broader and more explicit actions toward sustainability [66].

## 5. Conclusions

This study found moderate adherence to the Planetary Health Diet among Croatian university students, with significantly higher adherence among graduate students and those with greater physical activity. Higher diet quality, when adjusted for energy intake, was associated with lower environmental impacts, emphasizing the dual benefits of sustainable diets for human and planetary health. The study’s findings highlight that, despite the overall benefits of sustainable diets, the environmental impact can vary substantially based on specific dietary choices. Universities have a crucial role in shaping dietary behaviors through educational programs, the availability of sustainable food options, and behavior change strategies. Future research should investigate the complex relationships between diet, health, and environmental outcomes among students, considering lifestyle habits and campus food environments. To promote sustainable eating, practical interventions such as improved access to plant-based, local, and certified sustainable foods, the use of diet assessment tools, and educational programs are essential. These insights can guide university and national policies aimed at improving university students’ health and advancing sustainability goals.

## Figures and Tables

**Table 1:** Characteristics of 224 Croatian university students according to the Planetary Health Diet Index (PHDI) quartiles (N (%) or mean ± SD).

Variable	N (%)	*p*-Value	Planetary Health Diet Index	*p*-Value	Quartile 1 (20–45)	Quartile 2 (46–55)	Quartile 3 (56–65)	Quartile 4 (66–100)	*p*-Value
N	224 (100)		55.54 ± 13.30		57 (25)	72 (32)	57 (25)	38 (17)	0.019
Men	121 (54)	0.229	56.96 ± 11.10	0.086	23 (40)	41 (57)	38 (67)	19 (50)	0.038
Women	103 (46)		53.87 ± 15.48		34 (60)	31 (43)	19 (33)	19 (50)	
Age (years)	22.67 ± 2.19				22.44 ± 1.97	22.89 ± 2.33	22.67 ± 2.25	22.58 ± 2.18	0.704
Level of study									
Undergraduate	118 (53)	0.423	54.87 ± 12.50	0.188	45 (79)	37 (51)	27 (47)	20 (53)	0.004
Graduate	106 (47)		56.27 ± 14.16		12 (21)	35 (49)	30 (53)	18 (47)	
Nonsmokers	168 (75)	<0.001	55.83 ± 13.62	0.563	46 (81)	51 (71)	41 (72)	30 (79)	0.521
Smokers	56 (25)		54.64 ± 12.35		11 (19)	21 (29)	16 (28)	8 (21)	
Body mass index (kg/m^2^)	24.11 ± 3.50				23.29 ± 2.93	24.55 ± 3.86	24.08 ± 3.50	24.65 ± 3.50	0.181
Underweight	6 (3)	<0.001	53.33 ± 10.33	0.750	3 (5)	1 (1)	1 (2)	1 (2)	0.653
Normal weight	139 (62)		54.93 ± 13.12		37 (65)	43 (60)	39 (68)	20 (53)	
Overweight	64 (28)		56.56 ± 13.74		15 (26)	23 (32)	12 (21)	14 (37)	
Obesity	15 (7)		57.67 ± 14.74		2 (4)	5 (7)	5 (9)	3 (8)	
Physical activity level									
Low	45 (20)	<0.001	57.13 ± 11.32	0.101	11 (19)	15 (21)	12 (21)	7 (18)	0.001
Moderate	99 (44)		53.98 ± 13.98		35 (62)	37 (51)	16 (28)	11 (29)	
Vigorous	80 (36)		58.27 ± 12.56		11 (19)	20 (28)	29 (51)	20 (53)	
Energy intake (kcal/day)	2397.59 ± 1197.53				1951.67 ± 1233.90	2465.86 ± 1164.32	2692.25 ± 1135.83	2495.12 ± 1151.53	0.007
Mediterranean Diet Score (MDS)	4.00 ± 1.47				3.04 ± 1.27	3.96 ± 1.41	4.46 ± 1.39	4.82 ± 1.20	<0.001
Low adherence (MDS ≤ 4)	141 (63)	<0.001	51.63 ± 12.78	<0.001	49 (86)	49 (68)	29 (51)	14 (37)	<0.001
High adherence (MDS ≥ 5)	83 (37)		62.17 ± 11.45		8 (14)	23 (32)	28 (49)	24 (63)	
Dietary Inflammatory Index (DII)	1.07 ± 2.62				3.03 ± 2.13	1.25 ± 2.49	−0.19 ± 2.13	−0.31 ± 2.29	<0.001
Pro-inflammatory diet (DII > 0)	139 (62)	<0.001	51.12 ± 13.28	<0.001	53 (93)	47 (65)	25 (44)	14 (37)	<0.001
Anti-inflammatory diet (DII < 0)	85 (38)		62.76 ± 9.71		4 (7)	25 (35)	32 (14)	24 (63)	

Ordinal data were tested with a chi-squared test; numerical data with an ANOVA test.

**Table 2:** Energy-adjusted environmental impact indicators regarding demographic, lifestyle, and dietary characteristics of 224 Croatian university students (mean ± SD).

Variable	Carbon Footprint (kg CO_2_ eqv.)/1000 kcal	Water Footprint (m^3^)/1000 kcal	Ecological Footprint (m^2^·year)/1000 kcal
Total	2.22 ± 0.53	1.90 ± 0.34	4.83 ± 1.37
Men	2.33 ± 0.53	1.95 ± 0.34	5.13 ± 1.33
Women	2.11 ± 0.50	1.74 ± 0.34	4.47 ± 1.33
*p*-value	0.002	0.017	<0.001
Level of study			
Undergraduate	2.27 ± 0.43	1.93 ± 0.31	4.98 ± 1.22
Graduate	2.17 ± 0.57	1.87 ± 0.37	4.65 ± 1.50
*p*-value	0.137	0.186	0.077
Smoking status			
Smokers	2.25 ± 0.55	1.89 ± 0.34	4.81 ± 1.34
Nonsmokers	2.21 ± 0.53	1.90 ± 0.34	4.83 ± 1.38
*p*-value	0.643	0.812	0.920
BMI category			
Underweight	2.26 ± 0.29	1.86 ± 0.12	4.76 ± 0.66
Normal weight	2.22 ± 0.57	1.91 ± 0.37	4.83 ± 1.48
Overweight	2.15 ± 0.48	1.85 ± 0.30	4.67 ± 1.23
Obesity	2.49 ± 0.32	2.05 ± 0.21	5.43 ± 0.78
*p*-value	0.176	0.242	0.278
Physical activity level			
Low	2.18 ± 0.52	1.86 ± 0.34	4.72 ± 1.31
Moderate	2.27 ± 0.54	1.92 ± 0.35	4.90 ± 1.41
Vigorous	2.13 ± 0.51	1.88 ± 0.32	4.71 ± 1.32
*p*-value	0.216	0.632	0.587
Mediterranean Diet Score			
Low adherence (MDS ≤ 4)	2.24 ± 0.57	1.93 ± 0.37	4.87 ± 1.44
High adherence (MDS ≥ 5)	2.19 ± 0.45	1.85 ± 0.27	4.75 ± 1.24
*p*-value	0.462	0.052	0.535
Dietary Inflammatory Index			
Pro-inflammatory diet (DII > 0)	2.20 ± 0.60	1.91 ± 0.39	4.76 ± 1.48
Anti-inflammatory diet (DII < 0)	2.25 ± 0.41	1.88 ± 0.24	4.93 ± 1.11
*p*-value	0.492	0.448	0.333
Planetary Health Diet Index			
Quartile 1	2.46 ± 0.53	2.07 ± 0.36	5.43 ± 1.30
Quartile 2	2.20 ± 0.50	1.89 ± 0.28	4.73 ± 1.27
Quartile 3	2.15 ± 0.57	1.83 ± 00.39	4.66 ± 1.45
Quartile 4	2.01 ± 0.41	1.77 ± 0.25	4.35 ± 1.26
*p*-value	<0.001	<0.001	<0.001

Numerical data were tested for differences with a *t*-test between two groups and an ANOVA test between multiple groups. MDS, Mediterranean Diet Score; DII, Dietary Inflammatory Index.

**Table 3:** Associations between the Planetary Health Diet Index (PHDI) and energy-adjusted environmental impact indicators among 224 Croatian university students.

Environmental Impact Indicator		PDHI (z-Score)		
	β	95% CI		*p*-Value
Carbon footprint (kg CO_2_ equivalent)/1000 kcal				
Model crude	−7.24	−10.41	−4.06	<0.001
Model adjusted	−7.94	−11.17	−4.72	<0.001
Water footprint (m^3^)/1000 kcal				
Model crude	−12.96	−17.81	−8.11	<0.001
Model adjusted	−13.88	−18.76	−8.99	<0.001
Ecological footprint (m^2^·year)/1000 kcal				
Model crude	−2.80	−4.03	−1.56	<0.001
Model adjusted	−3.15	−4.41	−1.89	<0.001
PHDI Quartile 1				
Carbon footprint (kg CO_2_ equivalent)/1000 kcal				
Model crude	−3.14	−6.87	−0.59	0.097
Model adjusted	−3.76	−7.5	−0.25	0.048
Water footprint (m^3^)/1000 kcal				
Model crude	−6.08	−11.48	−0.69	0.028
Model adjusted	−6.93	−12.27	1.59	0.012
Ecological footprint (m^2^·year)/1000 kcal				
Model crude	−1.55	−3.05	−0.05	0.044
Model adjusted	−1.57	−3.06	−0.09	0.038
PHDI Quartile 2				
Carbon footprint (kg CO_2_ equivalent)/1000 kcal				
Model crude	0.80	−0.38	−1.98	0.180
Model adjusted	0.84	−0.47	−2.15	0.203
Water footprint (m^3^)/1000 kcal				
Model crude	1.70	−0.38	−3.79	0.108
Model adjusted	1.74	−0.51	4.00	0.127
Ecological footprint (m^2^·year)/1000 kcal				
Model crude	0.35	−0.11	0.81	0.138
Model adjusted	0.38	−0.14	0.90	0.148
PHDI Quartile 3				
Carbon footprint (kg CO_2_ equivalent)/1000 kcal				
Model crude	0.62	−0.57	1.81	0.303
Model adjusted	0.50	−0.70	1.69	0.408
Water footprint (m^3^)/1000 kcal				
Model crude	0.14	−1.62	1.90	0.875
Model adjusted	−0.05	−1.82	1.72	0.955
Ecological footprint (m^2^·year)/1000 kcal				
Model crude	0.21	−0.25	0.68	0.361
Model adjusted	0.14	−0.33	0.60	0.548
PHDI Quartile 4				
Carbon footprint (kg CO_2_ equivalent)/1000 kcal				
Model crude	−3.16	−8.23	1.90	0.213
Model adjusted	−4.72	−8.83	−0.61	0.025
Water footprint (m^3^)/1000 kcal				
Model crude	−7.25	−15.6	−1.11	0.087
Model adjusted	−7.98	−7.44	−5.42	0.005
Ecological footprint (m^2^·year)/1000 kcal				
Model crude	−1.78	−3.37	−0.19	0.029
Model adjusted	−2.79	−7.86	−0.27	0.056

Mixed-effects linear regression models with a random intercept for gender (men, women), level of study (undergraduate, graduate), body mass index classification, and level of physical activity.

**Table 4:** Associations between the Planetary Health Diet Index (PHDI) and the energy-adjusted usual intakes of macro- and micronutrients among 224 Croatian university students.

				PDHI (z-Score)		
	Mean	SD	β	95% CI		*p*-Value
Energy (kcal/d)	2397.59	1197.53	2.15	0.59	3.70	0.007
Protein (g/d)	99.48	61.85	−0.38	−0.69	−0.07	0.017
Animal protein (g/d)	71.06	54.23	−0.46	−0.71	−0.22	<0.001
Vegetable protein (g/d)	28.42	13.80	1.94	1.24	2.64	<0.001
Total fat (g/d)	114.46	64.96	0.14	−0.14	0.32	0.324
Saturated fat (g/d)	38.58	26.95	−1.67	−2.24	−1.10	<0.001
Monounsaturated fat (g/d)	41.57	23.31	0.80	0.36	1.23	<0.001
Polyunsaturated fat (g/d)	22.08	11.42	12.47	4.18	20.77	0.003
Omega-3 fatty acids (g/d)	1.24	0.93	19.26	13.92	24.61	<0.001
Omega-6 fatty acids (g/d)	0.82	0.67	−39.76	−55.4	−24.11	<0.001
Trans fatty acids (g/d)	2.13	1.35	−14.50	−20.70	−8.31	<0.001
Cholesterol (mg/d)	560.25	330.95	0.01	−0.01	0.03	0.211
Carbohydrates (g/d)	219.50	97.56	−0.10	−0.21	0.02	0.105
Dietary fibres (g/d)	21.45	10.95	2.59	1.99	3.19	<0.001
Alcohol (g/d)	13.08	18.02	0.36	0.06	0.66	0.020
Thiamine (mg/d)	2.10	1.36	7.67	−3.76	19.09	0.187
Riboflavin (mg/d)	2.62	1.36	4.34	−1.18	11.05	0.113
Niacin (mg/d)	24.36	13.66	0.49	−0.46	1.44	0.312
Folate (μg/d)	313.55	176.85	0.13	0.09	0.16	<0.001
Pyridoxine (mg/d)	3.76	2.18	1.17	−2.59	4.94	0.539
Cobalamin (μg/d)	5.69	4.09	−0.51	−2.53	1.51	0.622
Vitamin C (mg/d)	109.81	65.47	0.19	0.12	0.27	<0.001
Beta-carotene (mg/d)	3869.23	3074.19	0.04	0.02	0.06	<0.001
Retinol (RE/d)	412.37	237.49	−0.01	−0.02	0.07	0.518
Vitamin D (μg/d)	3.44	2.59	1.31	−1.22	3.84	0.308
Vitamin E (mg/d)	14.61	12.72	1.57	1.20	1.94	<0.001
Sodium (mg/d)	4512.87	3606.90	−0.01	−0.03	−1.96	0.027
Potassium (mg/d)	3485.65	1620.74	0.02	0.01	0.03	<0.001
Calcium (mg/d)	1056.49	748.60	−0.03	−0.02	0.11	0.684
Phosphorus (mg/d)	1415.71	980.39	−0.02	−0.00	−0.15	0.815
Magnesium (mg/d)	370.97	187.42	0.18	0.13	0.22	<0.001
Iron (mg/d)	19.79	9.60	2.68	1.71	3.64	<0.001
Zinc (mg/d)	14.29	8.98	0.88	−1.00	2.77	0.357
Selenium (mg/d)	36.39	20.83	0.21	−0.11	0.52	0.206
Iodine (μg/d)	55.42	37.42	−0.19	−0.42	0.04	0.109
Caffeine (mg/d)	195.64	190.46	−0.10	−0.21	0.01	0.084
Flavan-3-ols (mg/d)	37.71	40.99	0.15	0.04	0.26	0.010
Flavones (mg/d)	2.51	2.23	7.16	5.20	9.13	<0.001
Flavanols (mg/d)	132.10	97.72	0.16	0.11	0.21	<0.001
Flavonones (mg/d)	70.15	57.08	0.11	0.03	0.20	0.008
Anthocyanidins (mg/d)	23.72	19.70	0.23	0.07	0.38	0.004

Mixed-effects linear regression models with a random intercept for gender (men, women), level of study (undergraduate, graduate), body mass index classification, and level of physical activity.

**Table 5:** Associations between the Planetary Health Diet Index (PHDI), the energy-adjusted usual intakes of 16 PHDI component foods, the Mediterranean Diet Score, and the Dietary Inflammatory Index among 224 Croatian university students.

			PDHI (z-Score)			
	Mean	SD	β	95% CI		*p*-Value
Nuts and peanuts	22.23	27.21	1.38	1.03	1.74	<0.001
Legumes	53.02	53.19	1.41	1.43	2.40	0.005
Fruits	173.29	156.38	1.40	0.86	1.94	<0.001
Vegetables	214.60	132.54	3.56	2.34	4.78	<0.001
Whole grains	28.85	23.71	0.18	−0.31	0.67	0.465
Eggs	40.18	37.08	0.44	−0.19	1.07	0.167
Fish	47.34	45.05	0.90	0.41	1.40	<0.001
Tubers and potatoes	148.75	114.59	−0.03	−0.44	0.38	0.876
Dairy	389.78	283.52	−0.46	−0.69	−0.23	<0.001
Vegetable oils	41.57	23.31	0.90	0.43	1.37	<0.001
Dark green vegetable ratio	69.24	20.63	−0.02	−0.11	0.06	0.577
Red-to-orange vegetable ratio	30.32	20.20	0.06	−0.02	0.10	0.158
Red meat	158.19	147.83	−0.21	−0.56	0.14	0.234
Poultry and substitutes	86.88	60.77	−0.90	−1.42	−0.38	<0.001
Animal fats	38.58	26.95	−18.11	−24.47	−11.77	<0.001
Added sugars	46.48	43.32	−0.57	−0.84	−0.27	<0.001
Mediterranean Diet Score	4.00	1.47	4.29	3.22	5.36	<0.001
Dietary Inflammatory Index	1.07	2.62	−2.91	−3.51	−2.32	<0.001

Mixed-effects linear regression models with a random intercept for gender (men, women), level of study (undergraduate, graduate), body mass index classification, and level of physical activity.

## Data Availability

The data presented in this study are available on request from the corresponding author due to participants’ privacy and ethical reasons.

## Abbreviations

The following abbreviations are used in this manuscript:

NCDs	Non-Communicable Diseases
PHD	Planetary Health Diet
MD	Mediterranean Diet
IPAQ-LF	International Physical Activity Questionnaire—Long Form
FFQ	Food Frequency Questionnaire
MET	Metabolic Equivalent of Task
BMI	Body Mass Index
PHDI	Planetary Health Diet Index
MDS	Mediterranean Diet Score
DII	Dietary Inflammatory Index

## References

[1] Fanzo J., Davis C. (2019). Can diets be healthy, sustainable, and equitable?. Curr. Obes. Rep..

[2] Willett W., Rockström J., Loken B., Springmann M., Lang T., Vermeulen S., Garnett T., Tilman D., DeClerck F., Wood A. (2019). Food in the Anthropocene: The EAT–Lancet Commission on healthy diets from sustainable food systems. Lancet.

[3] Tilman D., Clark M. (2014). Global diets link environmental sustainability and human health. Nature.

[4] Li J., Pandian V., Davidson P.M., Song Y., Chen N., Fong D.Y.T. (2025). Burden and attributable risk factors of non-communicable diseases and subtypes in 204 countries and territories, 1990–2021: A systematic analysis for the Global Burden of Disease Study 2021. Int. J. Surg..

[5] Institute for Health Metrics and Evaluation (2024). Dietary Risks—Level 2 Risk.

[6] Steel N., Bauer-Staeb C.M.M., A Ford J., Abbafati C., Abdalla M.A., Abdelkader A., Abdi P., Zuñiga R.A.A., Abiodun O.O., Abolhassani H. (2025). Changing life expectancy in European countries 1990–2021: A subanalysis of causes and risk factors from the Global Burden of Disease Study 2021. Lancet Public Health.

[7] OECD, FAO (2023). Environmental Sustainability in Agriculture 2023.

[8] FAO (2024). Greenhouse Gas Emissions from Agrifood Systems—Global, Regional and Country Trends, 2000–2022.

[9] FAO (2024). Land Statistics 2001–2022—Global, Regional and Country Trends.

[10] FAO (2024). The State of Food and Agriculture 2024—Value-Driven Transformation of Agrifood Systems.

[11] FAO (2012). Sustainable Diets and Biodiversity—Directions and Solutions for Policy, Research and Action.

[12] Cacau L.T., De Carli E., de Carvalho A.M., Lotufo P.A., Moreno L.A., Bensenor I.M., Marchioni D.M. (2021). Development and validation of an index based on EAT-Lancet recommendations: The Planetary Health Diet Index. Nutrients.

[13] Aleksandrowicz L., Green R., Joy E.J.M., Smith P., Haines A. (2016). The impacts of dietary change on greenhouse gas emissions, land use, water use, and health: A systematic review. PLoS ONE.

[14] Raghoebar S., Mesch A., Gulikers J., Winkens L.H.H., Wesselink R., Haveman-Nies A. (2024). Experts’ perceptions on motivators and barriers of healthy and sustainable dietary behaviors among adolescents: The SWITCH project. Appetite.

[15] Jurado-Gonzalez P., López-Toledo S., Bach-Faig A., Medina F.-X. (2025). Barriers and enablers of healthy eating among university students in Oaxaca de Juarez: A mixed-methods study. Nutrients.

[16] Almoraie N.M., Alothmani N.M., Alomari W.D., Al-Amoudi A.H. (2025). Addressing nutritional issues and eating behaviours among university students: A narrative review. Nutr. Res. Rev..

[17] Pfeifer D., Rešetar J., Šteković M., Czlapka-Matyasik M., Verbanac D., Gajdoš Kljusurić J. (2023). Diet quality and its association with lifestyle and dietary behaviors among Croatian students during two COVID-19 lockdowns. Foods.

[18] Mieziene B., Burkaite G., Emeljanovas A., Tilindiene I., Novak D., Kawachi I. (2022). Adherence to Mediterranean diet among Lithuanian and Croatian students during COVID-19 pandemic and its health behavior correlates. Front. Public Health.

[19] Pavičić Žeželj S., Dragaš Zubalj N., Fantina D., Krešić G., Kenđel Jovanović G. (2019). Adherence to Mediterranean diet in University of Rijeka students. Paediatr. Croat..

[20] Tayhan F., Helvacı G. (2025). Evaluation of university students’ Mediterranean diet quality and sustainable eating behaviors: A cross-sectional study. Int. J. Environ. Health Res..

[21] Yolcuoğlu İ.Z., Kızıltan G. (2022). Effect of nutrition education on diet quality, sustainable nutrition and eating behaviors among university students. J. Am. Nutr. Assoc..

[22] Pınarlı Falakacılar Ç., Yücecan S. (2024). The impact of sustainability courses: Are they effective in improving diet quality and anthropometric indices?. Nutrients.

[23] Telleria-Aramburu N., Bermúdez-Marín N., Rocandio A.M., Telletxea S., Basabe N., Rebato E., Arroyo-Izaga M. (2022). Nutritional quality and carbon footprint of university students’ diets: Results from the EHU12/24 study. Public Health Nutr..

[24] Arrazat L., Nicklaus S., de Lauzon-Guillain B., Marty L. (2024). Behavioural determinants of healthy and environmentally friendly diets in French university students. Appetite.

[25] Arrazat L., Nicklaus S., de Lauzon-Guillain B., Marty L. (2023). Identification of three dietary groups in French university students and their associations with nutritional quality and environmental impact. Front. Nutr..

[26] Mortaş H., Navruz-Varlı S., Bilici S. (2024). Adherence to the Planetary Health Diet and its association with diet quality in the young adult population of Türkiye: A large cross-sectional study. Nutrients.

[27] O’Leary M., Mooney E., McCloat A. (2025). The relationship between nutrition knowledge and dietary intake of university students: A scoping review. Dietetics.

[28] Craig C.L., Marshall A.L., Sjöström M., Bauman A.E., Booth M.L., Ainsworth B.E., Pratt M., Ekelund U.L., Yngve A., Sallis J.F. (2003). International physical activity questionnaire: 12-country reliability and validity. Med. Sci. Sports Exerc..

[29] Willett W.C., Sampson L., Stampfer M.J., Rosner B., Bain C., Witschi J., Hennekens C.H., Speizer F.E. (1985). Reproducibility and validity of a semiquantitative food frequency questionnaire. Am. J. Epidemiol..

[30] Kaić-Rak A., Antonić K. (1990). Tablice o Sastavu Namirnica i Pića.

[31] National Food Institute, Technical University of Denmark (2022). Food Data (Version 4.2).

[32] U.S. Department of Agriculture (2019). Agricultural Research Service.

[33] Sofi F., Cesari F., Abbate R., Gensini G.F., Casini A. (2020). Adherence to Mediterranean diet and health status: Meta-analysis. BMJ.

[34] Dernini S., Berry E.M., Serra-Majem L., La Vecchia C., Capone R., Medina F.X., Aranceta-Bartrina J., Belahsen R., Burlingame B., Calabrese G. (2017). Med Diet 4.0: The Mediterranean diet with four sustainable benefits. Public Health Nutr..

[35] Marx W., Veronese N., Kelly J.T., Smith L., Hockey M., Collins S., Trakman G.L., Hoare E., Teasdale S.B., Wade A. (2021). The dietary inflammatory index and human health: An umbrella review of meta-analyses of observational studies. Adv. Nutr..

[36] Shivappa N., Steck S.E., Hurley T.G., Hussey J.R., Hébert J.R. (2014). Designing and developing a literature-derived, population-based dietary inflammatory index. Public Health Nutr..

[37] Mertens E., Kaptijn G., Kuijsten A., van Zanten H.H.E., Geleijnse J.M., van ‘t Veer P. (2019). SHARP Indicators Database (Version 2) [Dataset].

[38] Petersson T., Secondi L., Magnani A., Antonelli M., Dembska K., Valentini R., Varotto A., Castaldi S. (2021). SU-EATABLE LIFE: A Comprehensive Database of Carbon and Water Footprints of Food Commodities.

[39] Willett W.C., Howe G.R., Kushi L.H. (1997). Adjustment for total energy intake in epidemiologic studies. Am. J. Clin. Nutr..

[40] Lombardo M. (2025). Assessing Gender and Age Differences in the Adoption of Sustainable Diets: Insights from an Intervention of the Mediterranean Diet. Sustainability.

[41] Alkazemi D. (2019). Gender differences in weight status, dietary habits, and health attitudes among college students in Kuwait: A cross-sectional study. Nutr. Health.

[42] Gil M., Rudy M., Stanisławczyk R., Duma-Kocan P., Żurek J. (2022). Gender differences in eating habits of Polish young adults aged 20–26. Int. J. Environ. Res. Public Health.

[43] Sprake E.F., Russell J.M., Cecil J.E., Cooper R.J., Grabowski P., Pourshahidi L.K., Barker M.E. (2018). Dietary patterns of university students in the UK: A cross-sectional study. Nutr. J..

[44] Valli C., D’Addezio L., Rosi A. (2022). Educational level and diet quality: A systematic review. Nutrients.

[45] Alruwaili A., King J.A., Deighton K., Kelly B.M., Liao Z., Innes A., Henson J., Yates T., Johnson W., Thivel D. (2024). The association of smoking with different eating and dietary behaviours: A cross-sectional analysis of 80,296 United Kingdom adults. Addiction.

[46] Lonati E., Cazzaniga E., Adorni R., Zanatta F., Belingheri M., Colleoni M., Riva M.A., Steca P., Palestini P. (2024). Health-Related Lifestyles among University Students: Focusing on Eating Habits and Physical Activity. Int. J. Environ. Res. Public Health.

[47] Macit-Çelebi M.S., Bozkurt O., Kocaadam-Bozkurt B., Köksal E. (2023). Evaluation of sustainable and healthy eating behaviors and adherence to the Planetary Health Diet Index in Turkish adults: A cross-sectional study. Front. Nutr..

[48] Cacau L.T., Benseñor I.M., Goulart A.C., Cardoso L.O., Lotufo P.A., Moreno L.A., Marchioni D.M. (2021). Adherence to the Planetary Health Diet Index and obesity indicators in the Brazilian Longitudinal Study of Adult Health (ELSA-Brasil). Nutrients.

[49] Kocaadam-Bozkurt B., Bozkurt O. (2023). Relationship between adherence to the Mediterranean diet, sustainable and healthy eating behaviors, and awareness of reducing the ecological footprint. Int. J. Environ. Health Res..

[50] Seconda L., Egnell M., Julia C., Touvier M., Hercberg S., Pointereau P., Lairon D., Allès B., Kesse-Guyot E. (2020). Association between sustainable dietary patterns and body weight, overweight, and obesity risk in the NutriNet-Santé prospective cohort. Am. J. Clin. Nutr..

[51] Marchioni D.M., Cacau L.T., De Carli E., Carvalho A.M., Rulli M.C. (2022). Low adherence to the EAT-Lancet sustainable reference diet in the Brazilian population: Findings from the National Dietary Survey 2017–2018. Nutrients.

[52] Smith L., Jacob L., Firth J. (2023). Dietary patterns and obesity in young adults: A systematic review. Obes. Rev..

[53] Asghari G., Mirmiran P., Yuzbashian E., Azizi F. (2017). A systematic review of diet quality indices in relation to obesity. Br. J. Nutr..

[54] Camprodon-Boadas P., Gil-Dominguez A., De la Serna E., Sugranyes G., Lázaro I., Baeza I. (2025). Mediterranean diet and mental health in children and adolescents: A systematic review. Nutr. Rev..

[55] Li X., Chen M., Yao Z., Zhang T., Li Z. (2022). Dietary inflammatory potential and the incidence of depression and anxiety: A meta-analysis. J. Health Popul. Nutr..

[56] Schwingshackl L., Hoffmann G., Lampousi A.M. (2017). Adherence to Mediterranean diet and risk of cancer: An updated systematic review and meta-analysis. Nutrients.

[57] Tang H., Zhang X., Luo N., Huang J., Yang Q., Lin H., Lin M., Wu S., Wen J., Hong J. (2025). Temporal trends in the Planetary Health Diet Index and its association with cardiovascular, kidney, and metabolic diseases: A comprehensive analysis from global and individual perspectives. J. Nutr. Health Aging.

[58] Clark M.A., Springmann M., Hill J., Tilman D. (2020). Multiple health and environmental impacts of foods. Proc. Natl. Acad. Sci. USA.

[59] Springmann M., Godfray H.C.J., Rayner M., Scarborough P. (2018). Analysis and valuation of the health and climate change co-benefits of dietary change. Proc. Natl. Acad. Sci. USA.

[60] Ye Y.X., Geng T.T., Zhou Y.F., He P., Zhang J.J., Liu G., Willett W., Pan A., Koh W.P. (2023). Adherence to a Planetary Health Diet, environmental impacts, and mortality in Chinese adults. JAMA Netw. Open.

[61] Grech A., Howse E., Boylan S. (2020). A scoping review of policies promoting and supporting sustainable food systems in the university setting. Nutr. J..

[62] Franchini C., Biasini B., Rosi A., Scazzina F. (2023). Best practices for making the university campus a supportive environment for healthy and sustainable diets. Curr. Opin. Environ. Sci. Health.

[63] Graça J., Campos L., Guedes D., Roque L., Brazão V., Truninger M., Godinho C. (2023). How to enable healthier and more sustainable food practices in collective meal contexts: A scoping review. Appetite.

[64] Harrison L., Herrmann A., Quitmann C., Stieglbauer G., Zeitz C., Franke B., Danquah I. (2024). Effects of a cafeteria-based sustainable diet intervention on the adherence to the EAT-Lancet planetary health diet and greenhouse gas emissions of consumers: A quasi-experimental study at a large German hospital. Nutr. J..

[65] Aguilos M., Leggett Z., Jeffries S., Lupek M., Ardon M. (2025). University students’ ecological footprint and lifestyle changes: Awareness vs. action. Educ. Sci..

[66] Springmann M., Wiebe K., Mason-D’Croz D., Sulser T.B., Rayner M., Scarborough P. (2018). Health and nutritional aspects of sustainable diet strategies and their association with environmental impacts: A global modelling analysis with country-level detail. Lancet Planet. Health.

